# Ablação Septal Alcoólica no Brasil: Insights do Registro BRASA

**DOI:** 10.36660/abc.20250312

**Published:** 2025-07-08

**Authors:** Maria Antonieta Albanez A. de Medeiros Lopes, Mayara Viana, Júlia Nóbrega, Heitor N. Albanez A. de Medeiros, Gláucia Maria Moraes de Oliveira

**Affiliations:** 1 Real Hospital Português Recife PE Brasil Real Hospital Português, Recife, PE – Brasil; 2 Hospital São Marcos Recife PE Brasil Hospital São Marcos, Recife, PE – Brasil; 3 Hospital Universitário da Universidade Federal do Maranhão São Luís MA Brasil Hospital Universitário da Universidade Federal do Maranhão, São Luís, MA – Brasil; 4 Universidade Federal do Rio de Janeiro Rio de Janeiro RJ Brasil Universidade Federal do Rio de Janeiro, Rio de Janeiro, RJ – Brasil

**Keywords:** Intervenção Coronária, Ablação Septal Alcoólica, Cardiomiopatia Hipertrófica Obstrutiva

O Registro Multicêntrico Brasileiro de Ablação Septal Alcoólica (Registro BRASA), publicado na ABC Cardiol, representa uma contribuição fundamental para a compreensão da segurança, eficácia e aplicabilidade da Ablação Septal Alcoólica (ASA) no Brasil – um país com disparidades na saúde e uma alta prevalência de doenças cardiovasculares.^1^ Este registro fornece dados sem precedentes sobre os resultados da ASA em centros brasileiros, reforçando seu papel como uma alternativa viável à miotomia cirúrgica na cardiomiopatia hipertrófica obstrutiva (CMHO).

A cardiomiopatia hipertrófica (CMH) é um distúrbio cardíaco hereditário comum, historicamente associado à alta morbidade e mortalidade.^2^ No entanto, avanços em imagem diagnóstica, terapias farmacológicas e técnicas intervencionistas transformaram a CMH em uma condição tratável, com muitos pacientes agora alcançando expectativa de vida quase normal.^3^ Nas últimas duas décadas, as taxas de mortalidade diminuíram dramaticamente – de 6% para 0,5% ao ano – em grande parte devido à estratificação precoce de risco, desfibriladores *cardioverter* implantáveis e terapias de redução septal.^3^

Apesar desses avanços, as disparidades regionais persistem. Uma série histórica brasileira (2010–2020) revelou maior mortalidade relacionada à CMH no Nordeste e Sudeste, particularmente entre homens brancos e pardos com mais de 40 anos.^4^ Esses achados destacam a necessidade de protocolos padronizados para melhorar o diagnóstico precoce e o acesso ao tratamento – uma lacuna que o Registro BRASA busca abordar.

Para pacientes com CMHO sintomática refratária à terapia médica, a redução septal continua sendo a pedra angular do tratamento.^5,6^ Existem duas abordagens principais: a miotomia cirúrgica (o padrão ouro) oferece redução imediata e durável do gradiente, mas requer centros altamente especializados; e a ASA, uma alternativa menos invasiva, induz infarto controlado do septo hipertrófico, levando a remodelação gradual e alívio dos sintomas.^5,6^ Embora a miotomia seja preferida para pacientes mais jovens com anatomia complexa, desde sua introdução em 1995 por Sigwart et al., a ASA emergiu como uma alternativa atraente tanto para pacientes quanto para médicos.^7^ O número de procedimentos realizados aumentou rapidamente, superando o número de cirurgias realizadas anualmente em todo o mundo. Essa mudança foi impulsionada por sua natureza minimamente invasiva e resultados semelhantes a curto e médio prazo em comparação com procedimentos cirúrgicos em centros de excelência, como evidenciado por coortes de pacientes, registros e meta-análises, embora ensaios randomizados comparando as duas intervenções sejam escassos.^8^ No entanto, preocupações permanecem quanto às taxas mais altas de bloqueio átrio ventricular total (10–15%) e potencial arritmogênico devido à cicatrização septal. Vale ressaltar que o fator determinante para ter bons resultados com ambos os procedimentos é a experiência dos centros.^5,6^

O Registro BRASA incluiu um total de 41 pacientes realizados em quatro centros de referência terciária no Brasil. A idade média foi de 66,4 anos e 73% eram mulheres. Na linha de base, 93,2% estavam na classe NYHA III/IV ou classe CCS III/IV, com uma fração de ejeção do ventrículo esquerdo média de 66,4% e um gradiente médio do trato de saída do ventrículo esquerdo (TSVE) de 88,4 mmHg. Aos 12 meses, 92,8% melhoraram para NYHA I/II ou CCS I/II (p < 0,01). O gradiente médio do TSVE diminuiu de 88,4 mmHg para 27,0 mmHg (p = 0,003), e a espessura do septo interventricular (SIV) foi reduzida de 19,3 mm para 14,7 mm (p = 0,048). Os respondedores tiveram gradientes basais mais baixos (73,4 vs. 112,6 mmHg, p = 0,04) e menos hospitalizações (21,1% vs. 82,4%, p = 0,04). O bloqueio atrioventricular completo ocorreu em 16,7% dos pacientes, com 4,8% necessitando de implantação de marca-passo permanente. Nenhuma mortalidade foi observada após um seguimento médio de 394 dias, e 78,4% permaneceram na classe funcional I/II na última avaliação médica presencial.

Os resultados do registro BRASA são encorajadores. A ASA demonstrou ser um tratamento seguro e eficaz para aliviar os sintomas da CMHO), com uma redução significativa no gradiente de pressão do TSVE e na espessura do SIV. Notavelmente, 73% dos pacientes apresentaram uma resposta positiva ao procedimento, com melhora na classe funcional NYHA e redução nas hospitalizações durante o acompanhamento. O estudo também identificou um gradiente basal do TSVE inferior a 105 mmHg como um potencial preditor de resultados favoráveis. Esse achado destaca a importância da seleção cuidadosa de pacientes para otimizar os resultados do procedimento. Finalmente, a ASA provou ser segura, com baixas taxas de complicações e nenhuma mortalidade observada.

A técnica da ASA melhorou nas últimas décadas, incorporando ecocardiografia guiada por contraste e volumes de injeção de álcool menores, o que reforça a necessidade de discussão e apresentação de tais resultados à comunidade médica de cardiologia.^8–10^

Apesar dos avanços, o subdiagnóstico e os encaminhamentos atrasados persistem devido ao acesso limitado a centros especializados em CMH e à heterogeneidade na infraestrutura de saúde regional.^4^ O Registro BRASA sublinha a importância de equipes multidisciplinares de CMH para orientar decisões de tratamento, técnicas de ASA guiadas por contraste para minimizar complicações e registros de longo prazo para acompanhar resultados em populações diversas.

A conclusão deste manuscrito é que o Registro BRASA fornece insights valiosos sobre o papel da ASA no manejo da CMHO brasileira, demonstrando alívio significativo dos sintomas e redução do gradiente com um perfil de segurança favorável. Embora a miotomia cirúrgica permaneça preferida para casos complexos, a ASA oferece uma alternativa menos invasiva para pacientes cuidadosamente selecionados.

Avançando, expandir o acesso a terapias de redução septal e implementar registros nacionais de CMH será crucial para reduzir disparidades e melhorar os resultados em todo o país. O Registro BRASA marca um passo importante nessa direção, alinhando os dados brasileiros com padrões globais de atendimento à CMH.
