# Supplementary material for: Ablação Septal Alcoólica no Brasil: Insights do Registro BRASA
Source: Arq Bras Cardiol. 2025 Jul 8;122(6):e20250312. [Article in Portuguese] doi: 10.36660/abc.20250312 (PMC12269906; doi:10.36660/abc.20250312)
Supplement: Translation

# Translation: Alcohol Septal Ablation in Brazil: Insights from the BRASA Registry

**DOI:** 10.36660/abc.20250312i

**Authors:** Maria Antonieta Albanez A. de Medeiros Lopes, Mayara Viana, Júlia Nóbrega, Heitor N. Albanez A. de Medeiros, Gláucia Maria Moraes de Oliveira

**Affiliations:** 1 Recife PE Brazil Real Hospital Português, Recife, PE – Brazil; 2 Recife PE Brazil Hospital São Marcos, Recife, PE – Brazil; 3 São Luís MA Brazil Hospital Universitário da Universidade Federal do Maranhão, São Luís, MA – Brazil; 4 Rio de Janeiro RJ Brazil Universidade Federal do Rio de Janeiro, Rio de Janeiro, RJ – Brazil

**Keywords:** Coronary Intervention, Alcohol Septal Ablation, Hypertrophic Obstructive Cardiomyopathy

The Brazilian Multicenter Registry of Alcohol Septal Ablation (BRASA Registry), published in ABC Cardiol in 2025, represents a pivotal contribution to understanding the safety, efficacy, and real-world applicability of Alcohol Septal Ablation (ASA) in Brazil—a country marked by healthcare disparities and a high burden of cardiovascular diseases.^1^ This registry provides unprecedented data on ASA outcomes in Brazilian centers, reinforcing its role as a viable alternative to surgical myectomy in hypertrophic obstructive cardiomyopathy (HOCM).

Hypertrophic cardiomyopathy (HCM) is a common inherited cardiac disorder, historically associated with high morbidity and mortality.^2^ However, advances in diagnostic imaging, pharmacological therapies, and interventional techniques have transformed HCM into a treatable condition, with many patients now achieving near-normal life expectancy.^3^ Over the past two decades, mortality rates have declined dramatically—from 6% to 0.5% per year—largely due to early risk stratification, implantable cardioverter-defibrillators, and septal reduction therapies.^3^

Despite these advancements, regional disparities persist. A Brazilian historical series (2010–2020) revealed higher HCM-related mortality in the Northeast and Southeast, particularly among white and mixed-race (pardo) men over 40 years.^4^ These findings highlight the need for standardized protocols to improve early diagnosis and treatment access, a gap that the BRASA Registry seeks to address.

For patients with symptomatic HOCM refractory to medical therapy, septal reduction remains the cornerstone of treatment.^5,6^ Two primary approaches exist: surgical myectomy (the gold standard) offers immediate and durable gradient reduction but requires highly specialized centers; and ASA, a less invasive alternative, induces controlled infarction of the hypertrophic septum, leading to gradual remodeling and symptom relief.^5,6^ While myectomy is preferred for younger patients with complex anatomy, since its introduction in 1995 by Sigwart et al., ASA has emerged as an attractive alternative for both patients and physicians.^7^ The number of procedures performed rapidly increased, surpassing the number of surgeries performed annually worldwide. This shift was driven by its minimally invasive nature and similar short- and medium-term outcomes compared to surgical procedures in centers of excellence, as evidenced by patient cohorts, registries, and meta-analyses, though randomized trials comparing the two interventions are lacking.^8^ However, concerns remain regarding higher rates of complete heart block (10–15%) and potential arrhythmogenicity from septal scarring It is worth noting that the determinant factor for having good results with both procedures is the experience of centers.^5,6^

The BRASA Registry included a total of 41 patients performed in four tertiary referral centers in Brazil. The median age was 66.4 years, and 73% were women. At baseline, 93.2% were in NYHA class III/IV or CCS class III/IV, with a mean left ventricular ejection fraction of 66.4% and a mean left ventricular outflow tract (LVOT) gradient of 88.4 mmHg. At 12 months, 92.8% improved to NYHA I/II or CCS I/II (p < 0.01). The mean LVOT gradient decreased from 88.4 mmHg to 27.0 mmHg (p = 0.003), and interventricular septum (IVS) thickness was reduced from 19.3 mm to 14.7 mm (p = 0.048). Responders had lower baseline gradients (73.4 vs. 112.6 mmHg, p = 0.04) and fewer hospitalizations (21.1% vs. 82.4%, p = 0.04). Complete atrioventricular block occurred in 16.7% of patients, with 4.8% requiring permanent pacemaker implantation. No mortality was observed after a median follow-up of 394 days, and 78.4% remained in functional class I/II at the last in-person medical evaluation.

The results from the BRASA registry are encouraging. ASA was shown to be a safe and effective treatment for alleviating HOCM symptoms, with a significant reduction in LVOT pressure gradient and IVS thickness. Notably, 73% of patients exhibited a positive response to the procedure, with improvement in NYHA functional class and a reduction in hospitalizations during follow-up. The study also identified a baseline LVOT gradient of less than 105 mmHg as a potential predictor of favorable outcomes. This finding highlights the importance of careful patient selection to optimize procedure results. Finally, the ASA proved to be safe, with low complication rates and no mortality observed.

The ASA technique has improved in recent decades, incorporating contrast-guided echocardiography and smaller alcohol injection volumes, which reinforces the need for discussion and presentation of such results to the cardiology medical community.^8–10^

Despite progress, underdiagnosis and delayed referrals persist due to limited access to specialized HCM centers and heterogeneity in regional healthcare infrastructure.^4^ The BRASA Registry underscores the importance of multidisciplinary HCM teams to guide treatment decisions, contrast-guided ASA techniques to minimize complications, and long-term registries to track outcomes in diverse populations.

This manuscript concludes that the BRASA Registry provides valuable insights into the role of ASA in Brazilian HOCM management, demonstrating significant symptom relief and gradient reduction with a favorable safety profile. While surgical myectomy remains preferred for complex cases, ASA offers a less invasive alternative for carefully selected patients.

Moving forward, expanding access to septal reduction therapies and implementing nationwide HCM registries will be crucial to reducing disparities and improving outcomes across the country. The BRASA Registry marks an important step in this direction, aligning Brazilian data with global standards in HCM care.
